# 1821. Time Until Definitive Grafting of Battlefield-Related Burn Injuries Is Associated with Burn Wound Infection

**DOI:** 10.1093/ofid/ofad500.1650

**Published:** 2023-11-27

**Authors:** Connor Wakefield, Matthew Geringer, Laveta Stewart, Faraz Shaikh, M Leigh Carson, Dan Lu, Leopoldo Cancio, Jennifer Gurney, David R Tribble, David R Tribble, John Kiley

**Affiliations:** Brooke Army Medical Center, San Antonio, Texas; Brooke Army Medical Center, San Antonio, Texas; Infectious Disease Clinical Research Program, Henry Jackson Foundation, Bethesda, Maryland; Census, Rockville, Maryland; Infectious Disease Clinical Research Program, Department of Preventive Medicine and Biostatistics, Uniformed Services University of the Health Sciences, Bethesda, MD, USA, Bethesda, MD; HJF, Rockville, Maryland; U.S. Army Institute of Surgical Research, San Antonio, Texas; U.S. Army Institute of Surgical Research, San Antonio, Texas; Uniformed Services University of the Health Sciences, Bethesda, Maryland; Uniformed Services University of the Health Sciences, Bethesda, Maryland; BAMC, San Antonio, Texas

## Abstract

**Background:**

With thermal injuries, infection is the leading cause of mortality after initial injury. We examined factors associated with development of skin and soft-tissue infections (SSTIs) in military personnel who sustained burns.

**Figure:**
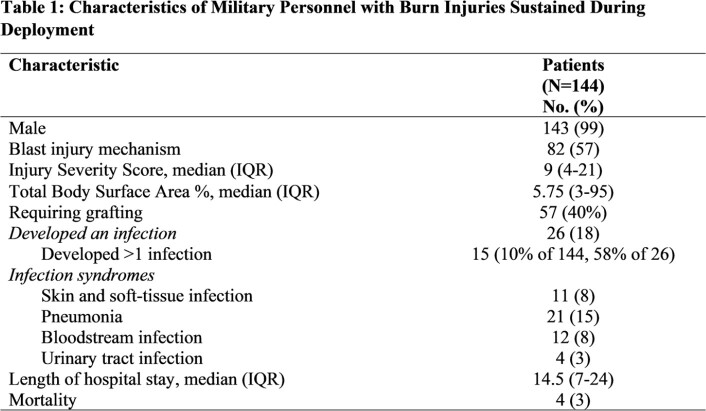

IQR= interquartile range

Multivariate Analysis of Factors Associated with Development of Skin and Soft-Tissue Infections

**Figure:**
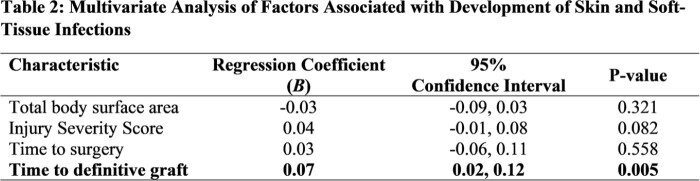

**Methods:**

Military personnel with burn injuries sustained during deployment (6/09-12/14) who required admission to the Burn Center at Brooke Army Medical Center were included in this analysis. Definitive grafting was defined as effective, successful wound closure noted in the surgical/wound care record. Infection outcomes were grouped by syndrome and comparative analysis using Fisher’s Exact test, Wilcoxon rank-sum test, and logistic regression were performed.

**Results:**

A total of 144 burn patients were included, of whom 143 (99%) were male with a median age of 24 years (Table 1). Approximately 57% sustained blast trauma and the median injury severity score (ISS) was 9. Total body surface area of the burn was a median of 5.75% (interquartile range [IQR]: 3-95%). Twenty-six (18%) burn patients developed an infection with 57% having ≥1 infection: 8% had SSTIs, 15% had pneumonia, 8% had bloodstream infections, and 3% had urinary tract infections. For the 57 (40%) patients who required grafting, the median days to definitive grafting was 9 (IQR: 6-56 days) for those without infections vs 35 days (IQR: 15-127 days) for those with ≥1 infection. Median hospital length of stay (LOS) was 14.5 days and 3% of patients died. On univariate analysis, time to surgery (p=0.032), duration of time until definitive graft (p< 0.001), increasing ISS (p< 0.001), and hospital LOS (p< 0.001) were associated with SSTI development, while injury mechanism (blast vs non-blast) was not significantly associated (p >0.05). On multivariate logistic analysis, increasing duration of time until definitive graft was associated with SSTI development when adjusted for ISS (regression coefficient: 0.07, 95% confidence interval: 0.02-0.12, p=0.005, Table 2), indicating a weak correlation.

**Conclusion:**

A longer time until definitive graft in wounded military personnel with burn injuries was associated with the development of SSTIs. Studies examining medical and surgical management strategies are needed to mitigate impact of infectious outcomes and reduce the time to definitive grafting in these patients.

**Disclosures:**

**All Authors**: No reported disclosures

